# Percent Body Fat-Related Disparities of Serum Ferritin on the Risk of Lipid Metabolism Abnormalities in Children and Adolescents

**DOI:** 10.3390/ijerph192316235

**Published:** 2022-12-04

**Authors:** Xin He, Wenjing Wang, Zhenni Zhu, Jiajie Zang, Tong Liu, Yan Shi, Chen Fu

**Affiliations:** 1 Laboratory of Functional Medicine, Division of Chronic Non-Communicable Diseases and Injury, Shanghai Municipal Center for Disease Control and Prevention, Shanghai 200336, China; 2 Department of Nutrition Hygiene, Division of Health Risk Factors Monitoring and Control, Shanghai Municipal Center for Disease Control and Prevention, Shanghai 200336, China

**Keywords:** ferritin, dyslipidemia, percent body fat, children, adolescent

## Abstract

Objective: This study examined the association between serum ferritin and dyslipidemia in children and adolescents with different degrees of obesity. Method: In this multi-stage, stratified, randomized, sampling cross-section cohort study, demographic data were collected by questionnaire from 4320 children and adolescents (aged 6–17 years) in Shanghai, China. Anthropometric measures and percent body fat (PBF) were recorded. Serum lipid parameters were detected by an automatic biochemical method, and ferritin levels were measured by an automatic immunoassay. Results: Our results showed 70.6%, 13.9%, and 15.5% of participants had a healthy body fat, low fat, and overweight/obese, respectively. Increasing ferritin quartiles were independently associated with a greater hazard of dyslipidemia, especially in overweight/obese participants, and the OR (95% CI) was 3.01 (1.29–7.00), 3.58 (1.59–8.04), and 5.66 (2.57–12.46) across the ferritin quartiles after adjustment for confounders. Ferritin was only a predictive value for dyslipidemia in overweight/obese participants (AUC = 0.64) and was consistent in boys (AUC = 0.61) and girls (AUC = 0.68). The significant positive correlation between ferritin value and lipid abnormalities profiles (except for low HDL-C) mainly appeared in the overweight/obesity group. Conclusion: The results showed that serum ferritin can be considered an independent risk factor for dyslipidemia in children and adolescents with obesity. Highlights: Ferritin overload had a greater risk of dyslipidemia, especially in children and adolescents with overweight/obesity.

## 1. Introduction

With economic development and lifestyle changes, the worldwide prevalence of dyslipidemia has become more common, and dyslipidemia now occurs more in younger people [1]; these are considered to be important modifiable risk factors for cardiovascular metabolic diseases [2,3]. In China, the national prevalence of dyslipidemia in adults aged 18 and older has increased from 18.6% in 2002 to 40.4% in 2012 [4,5], imposing a huge challenge to prevent and control cardiovascular metabolic diseases. Additionally, the China Child and Adolescent Cardiovascular Health (CCACH) study provided the incidence of dyslipidemia in children and adolescents in China as high as 16% [6], which presents a hidden danger for the risk of cardiovascular metabolic diseases in adulthood [7,8].

Iron is a critical metal element for human survival, but excess iron as a strong pro-oxidant has been found to provoke diseases due to its role in pathological catalytic reactions or oxidative stress [9]. Serum ferritin is the main iron-storage protein in the liver, maintaining the safety and bioavailability of excess iron, which is correlated to iron stores and considered as a sensitive index to evaluate the iron status of the body [10]. Iron metabolism disorder may lead to inflammation and oxidative stress, which may lead to atherosclerosis and other cardiovascular metabolic diseases, such as central obesity [11], dyslipidemia [11,12,13], hypertension [14], insulin resistance, and diabetes [11,12,13,14,15]. In the past three decades, China has witnessed a sharp rise in obesity, which is the main factor driving the epidemic of metabolic syndrome (Mets) and cardiovascular diseases (CVD) [16]. Meanwhile, the increase in obesity-related inflammatory signaling has been linked to increased hepatic hepcidin expression, resulting in disturbed iron homeostasis and the increase in ferritin [17]. We hypothesized that obesity may promote dyslipidemia attributing by iron metabolism. Up to now, there have been few studies in this field, especially in the young in China.

Taking advantage of a large cohort of children and adolescents with comprehensive measures of body fat, serum ferritin, and lipid parameters, we aimed to investigate the effect of obesity based on percent body fat (PBF) on the association of ferritin levels and dyslipidemia among children and adolescents.

## 2. Method

### 2.1. Study Population

During May to June 2015, a total of 4320 school-aged students (6–17 years) were recruited from 60 schools of 16 districts in Shanghai, China through multi-stage stratified random sampling to represent children and adolescents in Shanghai. A detailed cohort description has been published previously [18]. We excluded individuals with incomplete questionnaires (*n* = 237), missing anthropometric measurements (*n* = 78), an absence of blood assessment (*n* = 47), and incomplete test data (*n* = 115). To exclude possible acute inflammation, individuals with hypersensitive C-reactive protein (hs-CRP) ≥ 10 mg/L (*n* = 24) were also excluded. Finally, a total of 3819 children and adolescents (1897 boys and 1922 girls) participated in the final analysis. This study received approval from the Medical Ethics Committee of the Shanghai Municipal Center for Disease Control and Prevention (No. 2015-15, Shanghai, China). We obtained written informed consent from each participant and the participant’s guardian.

### 2.2. Anthropometric and Biochemical Measurements

Height and weight measurements were performed, with the participants wearing light clothing and no shoes. BMI was calculated as weight (kg) divided by the square of height (m^2^). Waist circumference was measured midway between the costal margin and iliac crest at the end of a normal expiration. The waist-to-height ratio (WHtR) was calculated as waist circumference (cm) divided by height (cm). The percent of body fat was automatically measured and calculated by a body-composition monitor (Tanita BC601, Tokyo, Japan). Blood pressure was measured three times after a quiet rest using an Omron HEM-7071 electronic sphygmomanometer (Omron Healthcare, Kyoto, Japan) and averaged. 10 mL of venous blood was obtained in the morning following an overnight fast. The serum concentrations of total cholesterol (TC), high-density lipoprotein cholesterol (HDL-C), low-density lipoprotein cholesterol (LDL-C), triglyceride (TG), and highly sensitive C-reactive protein (hs-CRP) were measured enzymatically using a HITACHI 7080 automatic biochemical analyzer with reagents from Wako Pure Chemical Industries, Ltd. (Tokyo, Japan). The concentration of serum ferritin was measured by immune-chemiluminescence with ACCESS 2 immunoanalyzer and the matching reagent (Beckman Coulter, Brea, CA, USA). The intra-assay CVs for TC, TG, LDL-C, HDL-C, hsCRP, and ferritin were 1.19%, 1.45%, 2.39%, 3.08%, 3.46%, and 6.57%, respectively, and the inter-assay CVs were 2.91%, 2.83%, 3.12%, 4.03%, 4.64%, and 7.88%, respectively. All clinical analyses were performed in the laboratory of the Shanghai Center for Disease Control and Prevention, which was certified by the Shanghai Clinical Testing Center (Shanghai, China).

### 2.3. Definition of Dyslipidemia

Dyslipidemia was defined according to the cut-off values of the National Heart, Lung, and Blood Institute of the US National Institutes of Health expert panel on integrated guidelines for cardiovascular health and risk reduction in children and adolescents [19,20]. Participants with any of the following were considered as dyslipidemia: (1) high TC: TC ≥ 200 mg/dL, (2) high TG: TG ≥ 110 mg/dL (<10 year old) or ≥130 mg/dL (≥ 10 year old), (3) high LDL-C: LDL-C ≥ 130 mg/dL, and (4) low HDL-C: HDL-C ≤ 40 mg/dL.

### 2.4. Potential Confounders

In this study, demographic and lifestyle information on age, gender, pubertal stage, and sedentary time was acquired by an interviewer-administered questionnaire, and dietary information was obtained by the food-frequency questionnaire (FFQ). The detailed assessment of diet and physical activities can be found in our previous study [18]. Pubertal stage was self-reported according to menstruation or spermatorrhea. Sedentary time was the sum of time spent using a computer, playing video games, reading, doing homework, and performing other sedentary activities. The dietary energy intake was estimated according to daily food and condiment consumption using the Chinese food composition database. Because there were few cases of smoking and drinking in the school-age population investigated, these two items were not included in the covariate analysis. Due to the inflammatory state may affect the blood lipid levels, those with hs-CRP ≥ 10 mg/L were excluded from the study.

### 2.5. Statistical Analysis

The general characteristics of the study participants were presented as the number (percentage, %) for categorical variables and the mean (SD) or median (IQR) for continuous variables. The gender- and age-standardized BMI Z-score and PBF Z-score were calculated using the standard deviation dispersion method, the Z-score = (measured value-mean value of age group)/standard deviation [21]. Then, we applied the PBF Z-score cutoff of less than −1 to identify low-fat participants and more than 1 to identify overweight/obesity participants, and the rest were healthy fat participants. Binary logistical regression models were used to calculate the odds ratios (ORs) and 95% CIs for dyslipidemia associated with gender-specific ferritin quartiles among all participants and across three obesity categories. The ORs (95% CI) were adjusted for age, gender, WHtR, pubertal stage, sedentary time, and energy intake. Subsequently, the correlation between ferritin quartiles and dyslipidemia was analyzed by binary logistic regression according to the BMI Z-score classification (with −1 and 1 as the range value). We did no imputation for missing data for the stratification variables. To normalize the distribution, the skewed variables of ferritin were log-transformed for further statistical analyses. ROC analysis was used to evaluate the performance of logarithmic ferritin in predicting dyslipidemia in different gender groups under different obesity categories to verify the consistency. The odds ratios of each lipid index abnormality in each 1-unit change in log-transformed ferritin increase were calculated using a binary logistic regression in the adjusted model controlling confounding factors. A two-sided *p* value of less than 0.05 was considered significant. All analyses were performed using Statistical Package for the Social Science (SPSS) (version 19.0, SPSS Inc. Chicago, IL, USA). All graphs were created using Prism version 5 (GraphPad Software Inc., San Diego, CA, USA).

## 3. Results

### 3.1. Characteristics of Participants

In this study, the PBF of girls was obviously higher than that of boys (23.11% ± 8.20% vs. 18.41% ± 9.66%). Boys had a higher level of serum ferritin than did girls (48.80 (33.20, 73.90) vs. 35.90 (23.08, 53.63) µg/L). A higher proportion of boys than girls had lipid abnormalities (high TG: 6.8% vs. 5.8%; low HDLC: 2.5% vs. 1.2%; high LCDC: 3.9% vs. 3.3%; dyslipidemia: 12.7% vs. 11.6%). In this study, 2686 participants (70.6%) were classified as having a healthy percentage body fat (PBF), 530 (13.9%) were within the low-fat range, while 591 (15.5%) of the participants were classified as overweight/obesity according to age- and gender-specific PBF Z-score cutoffs (Table 1).

The PBF Z-score cutoff of less than −1 was identified as low fat, of more than 1 was identified as overweight/obesity, and the rest were healthy fat.

Pubertal stage is expressed as the effective percentage of spermatorrhea for boys or menstrual cramps for girls. Continuous data are presented as mean ± SD or median (IQR); categorical data are presented as numbers (percentage).

### 3.2. Associations of Ferritin Quartiles with Dyslipidemia among Different PBF Categories

**Figure 1:**
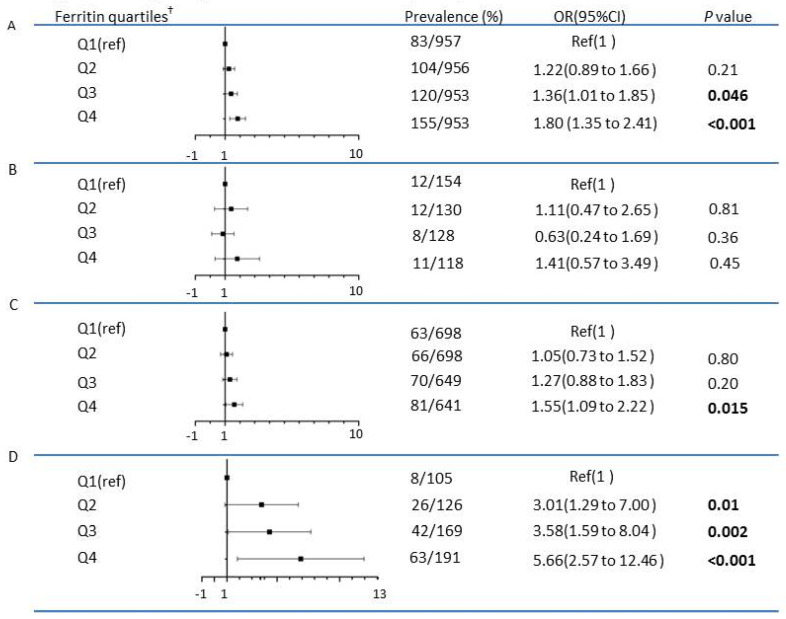
Adjusted odds ratio (OR) for dyslipidemia associated with sex-specific quartiles of ferritin among the overall participants and PBF-stratificated participants. (**A**) The overall participants (*n* = 3819) (**B**). Participants with low fat (PBF Z-scores < −1) (*n* = 530) (**C**). Participants with healthy fat (−1 ≤ PBF Z-scores ≤ 1 (*n* = 2686) (**D**). Participants with overweight and obese (PBF Z-scores > 1) (*n* = 591). * ORs (95% CIs) were adjusted for age, gender, WHtR, pubertal stage (pre-pubertal, pubertal), sedentary time, and energy intake. † Sex-specific quartiles of ferritin were defined among the overall participants and among participants with low fat, healthy fat, and overweight/obesity, separately. ORs shown in bold font are statistically significant at *p* < 0.05.

An increase in the sex-specific ferritin quartile was associated with an increased hazard of dyslipidemia. Overall, adjusted for covariates (including age, gender, WHtR, sedentary time, and energy intake), the hazard of dyslipidemia increased to 1.36 (1.01–1.85) and 1.80 (1.35–2.41) in participants with ferritin in quartiles 3 and 4, respectively, compared with participants with ferritin in the lowest quartile 1 among the overall participants (Figure 1). In the PBF Z-score stratified analysis, the positive association between ferritin quartiles and dyslipidemia was more pronounced in the overweight/obesity categories, and there was no association among participants with low body fat. Among participants with healthy body fat, compared with the lowest quartile, only the highest quartile of ferritin (quartile 4 vs. quartile 1) showed a risk of dyslipidemia, which was 1.55 (95% CI 1.09–2.22). Among participants with overweight/obesity, compared with the lowest quartile, all the other three quartiles of ferritin (quartile 2, 3, 4 vs. quartile 1) showed the risk of dyslipidemia, and the OR gradually increased with the increases of the quartile, which were 3.01 (1.29–7.00), 3.58 (1.59–8.04), and 5.66 (2.57–12.46) in quartile 2, 3 and 4, respectively. However, in the stratified analysis of BMI Z-score classification, the positive correlation between ferritin quartiles and dyslipidemia was not related in the overweight/obesity category.

ROC analysis was used to evaluate the predictive performance of per unit increase in log-transformed ferritin on dyslipidemia. Only the AUC (95% CI) in participants with overweight/obesity showed the predictive effect of ferritin on dyslipidemia, which was 0.64 (0.59–0.69). This predictive effect was consistent in boy and girl groups, and the predictive effect in girl groups was slightly higher than that in boy groups (0.68 (0.61–0.75) vs. 0.61 (0.54–0.68)).

### 3.3. Relationship between Ferritin and Lipid Abnormalities Profiles among Different PBF Categories

After controlling covariates, the comparative OR and 95% CI showed that lipid abnormality profiles exhibited different associations with log-transformed ferritin among different obesity categories (Table 2 and Table 3). The significant positive correlation between ferritin value and multiple lipid indexes abnormalities mainly appeared in the overweight/obesity group. The multivariable adjusted ORs of high TC, high TG, and high LCD-C were 23.96 (95% CI: 5.71–100.47), 9.67 (4.20–22.26), and 11.82 (3.45–40.51), respectively. In the healthy body fat group, the increase in each 1 unit log-transformed ferritin was positively correlated with high TG and high LDL-C (OR: 2.55, 95% CI: 1.35–4.83; OR: 3.65, 95% CI: 1.64–8.13). However, there was no significant correlation between ferritin and lipid abnormalities in the low-body-fat group. In this study, subjects with elevated ferritin did not show a significant correlation with adverse low HDL-C in all three body fat categories (all *p* > 0.05) (Table 3).

## 4. Discussion

This cohort study aimed to evaluate the correlation between serum ferritin and lipid abnormalities in Chinese children and adolescents, and the impact of PBF-based obesity. The results showed that ferritin levels were positively related to the prevalence of dyslipidemia as well as single lipid index abnormality, including high TC, high TG, and high LDL-C. These positive associations were more pronounced among participants with overweight/obesity than those with healthy and low fat levels. Meanwhile, ferritin levels had a predictive value on dyslipidemia in subjects with overweight/obesity, and this was consistent for boys and girls. These results were attributable to obesity based on PBF, suggesting that obesity and ferritin overload may have a synergistic effect on dyslipidemia in Chinese children and adolescents.

This study demonstrated a significant association in increased ferritin levels with higher lipid levels and the risk of dyslipidemia, independent of confounding factors, such as age, gender, and lifestyle factors, which is consistent with previous studies in several countries [12,22,23]. However, the mechanism driving the relationship of circulatory ferritin with lipid metabolism is not fully understood. One possible explanation is that excess iron, which can present as a high ferritin concentration, catalyzed the formation of free radicals and induced oxidative stress damage to cells and metabolic tissues, and has been proposed to contribute to some metabolic abnormalities [24], including non-alcohol hepatic steatosis and fibrosis [25], insulin resistance [26], and dyslipidemia [27]. In addition, circulating ferritin is not only an ideal parameter for iron storage but also commonly elevated in chronic inflammation [28]. Inflammatory cytokines could promote the expression of hepcidin and the increase in ferritin, and higher circulating ferritin would increase lipid peroxidation [29,30]. This suggests that elevated serum ferritin levels may be associated with disrupted lipid metabolism and may be an independent risk factor of CVD, which is worth investigating in future studies. It is worth noting that the iron metabolism–microbiome–lipid metabolism may be a new mechanism perspective. Iron is a critical nutrient for both mammals and microorganisms [31,32]. Only 5–15% of iron is absorbed, and the rest enters the colon where it is available to the gut microbiota [33]. Its abundance can affect the composition of gut microbiota [33], further affecting lipid metabolism and the development of metabolic diseases.

A cross-sectional study involving 1120 Irish adults found that elevated serum ferritin displays significant positive associations with serum triglycerides, with an increased but non-significant presentation of HDL-C among overweight/obesity participants [34]. Another cross-sectional study conducted in Guangzhou, China, found that among children with obesity attributed to BMI, ferritin levels in the dyslipidemia group were higher than those in the healthy fat group [35]. Other studies have also shown that ferritin is an independent risk factor for nonalcoholic fatty liver in children with obesity and in adolescents [36,37]. These findings suggested there is a synergistic effect of ferritin and obesity on abnormal lipid metabolism both in children and adults. However, most of these studies in children used BMI as the basis for obesity classification. Our study found that the positive correlation between ferritin and the risk of dyslipidemia was more evident in children and adolescents with obesity based on PBF than that in healthy and low-fat participants. Obesity is a low-grade chronic inflammation state. Oxidative stress and inflammation caused by obesity may contribute to lipid synthesis and metabolic disorders [38]. In addition, inflammatory factors secreted by visceral fat (such as IL-6) enter the portal vein circulation and flow directly to the liver, which may stimulate hepatic phospholipid synthesis and cause an increase in ferritin [39,40]. Therefore, metabolic inflammation based on excess fat may be the root cause of obesity and ferritin, increasing the risk of dyslipidemia. This may also be the reason why the results we found in PBF classification are not repeated in BMI classification.

These results have implications for the prevention of dyslipidemia in Chinese children and adolescents. In view of the rapid increase in obesity in children and adolescents, with the synergistic link between obesity on ferritin overload and dyslipidemia, monitoring the ferritin levels of individuals with obesity and intervening with lipid abnormalities are warranted and should be promoted. Meanwhile, there are several limitations of this study, which should be mentioned. First, this cross-sectional study is insufficient to draw a causal relationship between ferritin status and lipid metabolism abnormality in obesity. Second, the existence of other confounding factors, such as dietary iron sources and iron supplements, cannot be ruled out. Moreover, due to the limitations of investigation methods, puberty status is judged by menarche or spermatorrhea instead of by Marshall Tanner standards. Third, serum iron and total iron-binding capacity were not measured in conjunction with serum ferritin levels. Another limitation is the use of PBF only as an indicator of obesity among children and adolescents; more detailed measures of body-fat distribution may strengthen the findings.

## 5. Conclusions

In conclusion, ferritin levels were significantly positively correlated with major lipid parameters, in which obesity had a synergistic effect, suggesting that the relationship between iron metabolism and lipid metabolism is worthy of further study.

## Figures and Tables

**Table 1:** Characteristics of the study participants.

Characteristics	Boys (*n* = 1897)	Girls (*n* = 1922)
Age, years	11.55 ± 3.40	11.54 ± 3.42
6–10 years, *n* (%)	767 (50.8)	742 (49.2)
11–17 years, *n* (%)	1130 (48.9)	1180 (51.1)
PBF (%)	18.41 ± 9.66	23.11 ± 8.20
BMI (kg/m^2^)	19.54 ± 4.19	19.45 ± 4.78
WHtR	0.44 ± 0.06	0.44 ± 0.06
Sedentary time, h/d	3.3 ± 1.5	3.7 ± 1.4
Energy intake, kcal/d	2043.73 ± 638.88	2127.73 ± 646.60
Pubertal stage, *n* (%)	467(24.6)	968 (50.4)
Hs-CRP, mg/L	0.30 (0.10, 0.80)	0.20 (0.10, 0.50)
Serum ferritin (µg/L)	48.80 (33.20, 73.90)	35.90 (23.08, 53.63)
TC (mg/dL)	150.18 ± 27.55	155.67 ± 26.26
TG (mg/dL)	52.26 (38.09, 74.40)	56.69 (45.52, 78.83)
HDL-C (mg/dL)	59.39 ± 12.36	61.89 ± 12.08
LDL-C (mg/dL)	83.21 ± 23.05	85.85 ± 21.74
High TC (≥200 mg/dL), *n* (%)	95 (5.0)	103 (5.4)
High TG (≥100 mg/dL, 0–9 years; ≥130 mg/dL, 10–19 years), *n* (%)	129 (6.8)	111 (5.8)
Low HDLC (≤40 mg/dL), *n* (%)	48 (2.5)	24 (1.2)
High LDLC (≥130 mg/dL), *n* (%)	73 (3.9)	63 (3.3)
Dyslipidemia, *n* (%)	240 (12.7)	222 (11.6)
Obesity categories based on PBF		
Low body fat, *n*(%)	272 (14.4)	258 (13.5)
Healthy body fat, *n*(%)	1307 (69.2)	1379 (71.9)
Overweight/obesity, *n*(%)	311 (16.5)	280 (14.6)

Abbreviations: BMI, body mass index; PBF, percent body fat; WHtR, waist to height ratio; Hs-CRP, highly sensitive C reactive protein; TC, total cholesterol; TG, triglyceride; HDL-C, high-density lipoprotein cholesterol; and LDL-C, low-density lipoprotein cholesterol.

**Table 2:** Performance of serum ferritin in predicting dyslipidemia among participants with different PBF categories.

			Serum Ferritin †			
	Low Fat (PBF Z-Score < −1)		Healthy Fat (−1 ≤ PBF Z-Score ≤ 1)		Overweight/Obesity (PBF Z-Score > 1)	
Gender	AUC (95% CI)	*p*	AUC (95% CI)	*p*	AUC (95% CI)	*p*
All	0.50 (0.41 to 0.58)	0.92	0.53 (0.50 to 0.57)	0.07	**0.64** **(0.59 to 0.69)**	**<0.001**
Boys	0.46 (0.32 to 0.60)	0.53	0.53 (0.47 to 0.58)	0.30	**0.61** **(0.54 to 0.68)**	**0.004**
Girls	0.56 0.45 to 0.67)	0.37	0.55 (0.50 to 0.59)	0.07	**0.68** **(0.61 to 0.75)**	**<0.001**

† = log10-transformed variable. AUCs shown in bold font are statistically significant at *p* < 0.05.

**Table 3:** Adjusted linear regression analysis of relationship between serum ferritin and lipid abnormality parameters among participants with different PBF categories.

				Serum Ferritin †					
Characteristics	Low Fat (PBF Z-Score < −1)			Healthy Fat (−1 ≤ PBF Z-Score ≤ 1)			Overweight/Obesity (PBF Z-Score > 1)		
	Rate	OR (95% CI)	*p*	Rate	OR (95% CI)	*p*	Rate	OR (95% CI)	*p*
High TC	4.5%	2.00 (0.42 to 9.57)	0.39	5.1%	1.68 (0.89 to 3.19)	0.11	6.1%	**23.96** **(5.71 to 100.47)**	**<0.001**
High TG	2.8%	0.71 (0.10 to 5.03)	0.73	4.5%	**2.55** **(1.35 to 4.83)**	**0.004**	17.8%	**9.67** **(4.20 to 22.26)**	**<0.001**
Low HDLC	0.8%	-	-	1.7%	1.95 (0.67 to 5.63)	0.22	3.7%	0.41 (0.09 to 1.81)	0.24
High LDLC	2.1%	0.71 (0.10 to 5.03)	0.67	3.4%	**3.65** **(1.64 to 8.13)**	**0.001**	5.9%	**11.82** **(3.45 to 40.51)**	**<0.001**

Abbreviations: TC, total cholesterol; TG, triglyceride; HDL-C, high-density lipoprotein cholesterol; and LDL-C, low-density lipoprotein cholesterol. *p* value was analyzed using the chi-squared test to compare rates. ORs shown in bold font are statistically significant at *p* < 0.05. † = log10-transformed variable, and OR (95% CI) of low HDL-C levels in the low-fat group could not be estimated as cases were less than 5.

## Data Availability

The data presented in this study are available on request from the corresponding author. The data are not publicly available due to privacy of juveniles.

## Abbreviations

BMI: body mass index; PBF, percent body fat; WHtR, waist to height ratio; Hs-CRP, high sensitive C reactive protein; TC, total cholesterol; TG, triglyceride; HDL-C, high-density lipoprotein cholesterol; and LDL-C, low-density lipoprotein cholesterol.
